# In Silico Identification and Molecular Characterization of *Lentilactobacillus hilgardii* Antimicrobial Peptides with Activity Against Carbapenem-Resistant *Acinetobacter baumannii*

**DOI:** 10.3390/antibiotics14101004

**Published:** 2025-10-10

**Authors:** Amanda Appel, Lily Velazco, Adit B. Alreja, Kara LeClair, Aryaan P. Duggal, Isha Vashee, Aji Mary Taal, Norberto Gonzalez-Juarbe, Derrick E. Fouts

**Affiliations:** 1 Infectious Diseases, The J. Craig Venter Institute, Rockville, MD 20850, USA; 2 Human Genomic Medicine, The J. Craig Venter Institute, Rockville, MD 20850, USA; 3 Cell Biology & Molecular Genetics Department, University of Maryland, College Park, MD 20742, USAivashee@umd.edu (I.V.)

**Keywords:** bioinformatic discovery, antimicrobial peptides, class IIb bacteriocin, biofilm disruption, lactic acid bacteria, *Acinetobacter baumannii*, ESKAPE, antibiotic synergy

## Abstract

Background/Objectives: Biofilm formation by *Acinetobacter baumannii* contributes to its persistence in clinical settings and resistance to antibiotic treatment. This study aims to identify and characterize antimicrobials from lactic acid bacteria (LAB) using molecular and in silico approaches that can prevent and disrupt *A. baumannii* biofilms, assess their antimicrobial host range, and define their synergy with current antibiotics. Methods: Thirteen LAB isolates from the Human Microbiome Project were screened for anti-biofilm activity against *A. baumannii*. Conditioned media was further tested against six ESKAPE pathogens and three skin commensals. *Lentilactobacillus hilgardii* was selected for detailed study and antimicrobial peptide (AMP) prediction analysis due to limited toxicity toward commensals. In silico identified peptides were synthesized and tested individually and in combination with sub-MIC doses of an antibiotic. Results: Conditioned media from five LAB species (*Lentilactobacillus hilgardii*, *Lentilactobacillus buchneri*, *Ligilactobacillus ruminis*, *Limosilactobacillus fermentum*, and *Limosilactobacillus antri*) significantly inhibited *A. baumannii* biofilm formation and reduced biomass of mature biofilms. LAB-conditioned media also exhibited broad-spectrum activity against ESKAPE pathogens, though effects on commensals varied. Bioinformatically predicted AMPs from *L. hilgardii* inhibited planktonic *A. baumannii* growth but showed no direct biofilm activity even at high doses. However, AMPs were found to synergize with sub-MIC doses of meropenem against mature biofilms leading to decolonization. Conclusions: Our study provides a comprehensive platform for the discovery and characterization of AMPs and supports using commensal bacteria to reduce, prevent, and decolonize biofilms from pathogenic bacteria in community and nosocomial settings.

## 1. Introduction

Nosocomial infections, also known as healthcare-associated infections (HAIs), are infections that are acquired while receiving healthcare treatment in hospitals, long-term care facilities, or ambulatory care. These infections can affect patients as well as healthcare staff. A variety of pathogens can cause nosocomial infections, including bacteria, fungi, and viruses [1]. Common among these different groups of pathogens is their resistance to the more potent drugs available in healthcare facilities, providing these pathogens with an infectious niche [2]. Of these pathogens, bacteria are the most common causative agents of nosocomial infections. ESKAPE is an acronym used to describe six highly antibiotic-resistant bacterial pathogens that commonly cause nosocomial infections, which include *Enterococcus faecium*, *Staphylococcus aureus*, *Klebsiella pneumoniae*, *Acinetobacter baumannii*, *Pseudomonas aeruginosa*, and *Enterobacter* spp. [3]. Together, these pathogens have been shown to account for 42.2% of HAIs isolated from bloodstream infections, 65.3% from surgical site infections, and 75.6% from an intensive care unit, highlighting their prevalence and significance in the hospital environment [4,5,6].

While this study aims to curb the persistence of all ESKAPE pathogens, we focused on *A. baumannii* as our model organism. *Acinetobacter* spp. are Gram-negative, rod-shaped bacteria typically found in environmental microbiomes such as soil and water [7]. Despite their environmental niches, *A. baumannii* has been commonly detected and isolated from medical devices for several decades [8]. *Acinetobacter*-associated nosocomial infections manifest in a variety of ways, including meningitis, pneumonia, as well as wound, burn, and urinary tract infections [9]. Antibiotic resistance is a growing problem affecting many different types of bacteria, including *A. baumannii*, which are highly resistant to antibiotics largely employed in hospital settings, including but not limited to quinolones, penicillins, and aminoglycosides [10]. Of significance, many strains of *A. baumannii* are resistant to carbapenems (i.e., carbapenem-resistant *A. baumannii* [CRAb]), the antibiotic reserved for multidrug-resistant bacteria and considered to be a last resort antibiotic [11]. Carbapenems function by gaining access through outer membrane proteins (porins) and acylating penicillin-binding proteins, proteins responsible for maintaining peptidoglycan in the bacterial cell wall [12]. This high level of resistance has resulted in the CDC placing *A. baumannii* on its urgent threats list [13]. The increase in antibiotic-resistant strains and decrease in effectiveness of existing antibiotics raise the need for alternative antimicrobials.

Antimicrobial peptides (AMPs) are one such alternative to antibiotics to combat multidrug-resistant bacterial infections. AMPs encoded by lactic acid bacteria (LAB) are known as bacteriocins and are used by the producing strain to defend against niche-competing bacteria [14]. While there have been numerous reports of antimicrobial activity from LAB cell-free cultures (isolated from fermented foods) against Gram-positive and Gram-negative pathogens [15,16], the identity of the compound(s) responsible for said activity has been determined in only a handful of examples (e.g., Reuterin, Plantaricin, Helveticin, Lactacin) [17]. AMPs are also commonly produced by eukaryotic cells and commensal bacteria and can act as a form of antagonist, especially in the gut, by preventing colonization by pathogenic bacteria like *A. baumannii* and *P. aeruginosa* [18]. While hundreds of AMPs with antagonistic properties toward *A. baumannii* have been discovered, the vast majority of them are not derived from microbial sources [19]. Recent work has described anti-*A. baumannii* activity from various *Lactobacillus* spp. [20,21,22], but only one identified the molecule responsible for the antimicrobial activity [21], which was not an AMP. Synergy between LAB-conditioned media and antibiotics was effective at inhibiting the growth of MDR *A. baumannii* clinical isolates [20]. Here, we aim to investigate the tools employed by commensal bacteria, particularly those within the former *Lactobacillus* genus, in hopes of discovering novel antimicrobials for the inhibition of *A. baumannii* planktonic growth and biofilms.

## 2. Results

### 2.1. Conditioned Media Prevents Formation of and Disrupts Pre-Formed A. baumannii Biofilms

**Figure 1:**
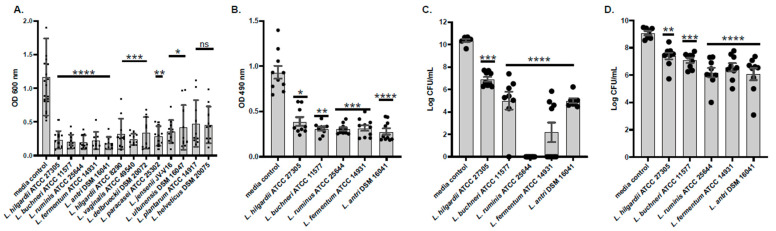
LAB-conditioned media impede *A. baumannii* biofilms and viability. Thirteen isolates from 12 different human commensal LAB species were tested against *A. baumannii* ABUH773 in biofilm assays, where *A. baumannii* and cell-free LAB-conditioned media were co-incubated statically at 37 °C. Biofilm integrity was determined after 24 h of coincubation, and then the biofilms were stained using 0.1% crystal violet (**A**). Assays to measure disruption of pre-formed *A. baumannii* biofilms by the six most significant biofilm-inhibiting supernatants (boldface, panel (**A**)) were conducted over the course of four days, with the original seeding, the refreshing of media the following day, and the introduction of cell-free LAB-conditioned media on the third day. The fourth day involved the fixing, staining, and washing of biofilms. Biofilms were stained with 0.1% crystal violet (**B**). Bacteria in supernatant viability was determined by CFU/mL measurements (**C**), as well as that bound to the plate (biofilm) viability (**D**). Viability data are reported as the Log CFU/mL of recovered bacteria. Non-parametric Kruskal–Wallis tests were employed to determine significance between MRS control and LAB supernatant experimental conditions in the 24 h biofilm assay. Experiments were performed in triplicate with mean values plotted and error bars representing standard deviations. Black dots are values of individual replicates. For the non-parametric Kruskal–Wallis tests, asterisks denote the level of significance observed: ns, not significant; * *p* ≤ 0.05, ** *p* ≤ 0.01, *** *p* ≤ 0.001, and **** *p* ≤ 0.0001.

Of the 13 human microbiome project (HMP) lactic acid bacteria (LAB) isolates that were tested for the ability to prevent *A. baumannii* biofilm formation (Table S1), only 5 reached a *p*-value of <0.0001 and were all unique species (Figure 1A). These five LAB species included *Lentilactobacillus hilgardii*, *Lentilactobacillus buchneri*, *Ligilactobacillus ruminis*, *Limosilactobacillus fermentum*, and *Limosilactobacillus antri*. These five species were further tested for their ability to disrupt pre-formed *A. baumannii* biofilms as well as their ability to reduce the viability of *A. baumannii* cells after exposure to LAB supernatants. Biofilm disruption was quantified by measuring changes in biomass percentage using crystal violet staining. When co-incubated with the five LAB supernatants, biofilm biomass decreased approximately 50% compared to the culture media control. (Figure 1B). Then, we tested the effect of the five LAB in mature biofilms. After a one-hour treatment, we collected the bacteria in the supernatant and the bacteria still attached to the plate. Conditioned media from *L. ruminis* showed the strongest inhibition, with a 10-log fold difference and complete eradication of planktonic *A. baumannii* dispersed from the biofilm. While no other species had this strong effect, two other species, including *L. fermentum* and *L. antri*, significantly reduced the viability of *A. baumannii* cells within biofilms. *L. ruminis* and *L. fermentum* were both able to attenuate planktonic *A. baumannii* growth with a *p*-value of <0.001. Viability of *A. baumannii* within the biofilm matrix was less attenuated by *L. hilgardii*- and *L. buchneri*-conditioned media (Figure 1C,D).

### 2.2. Determination of Susceptibility Range Within ESKAPEs and Known Skin Commensals

**Figure 2:**
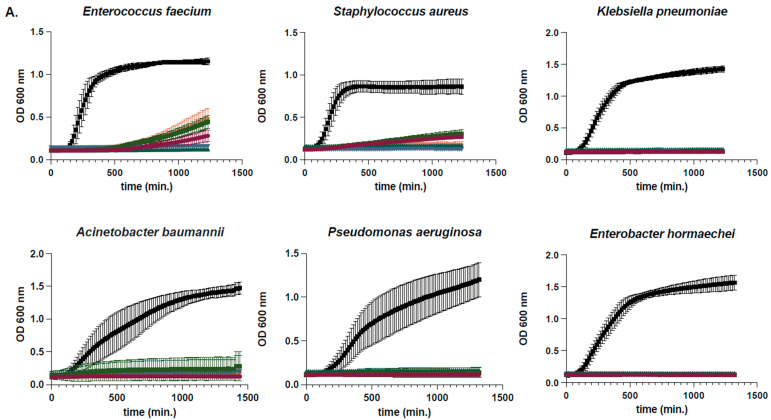

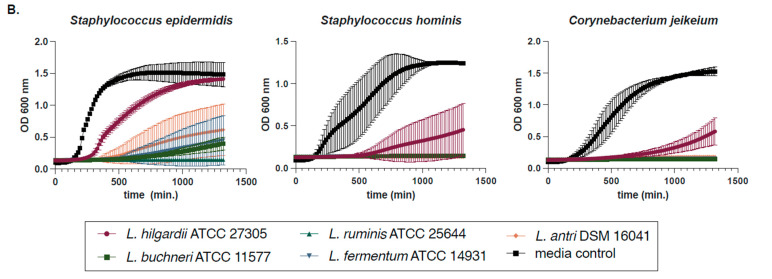
The effect of LAB-conditioned media on the planktonic growth of ESKAPE pathogens and skin commensal organisms. Bacterial growth in liquid media was measured by optical density at 600 nm wavelength for 24 h. An equal volume of cell-free supernatants from five LAB or media control was tested for inhibition of growth of six representative ESKAPE pathogen isolates (**A**) and three representative skin commensal isolates (**B**). Colors indicate different LAB supernatants or media control (see Key). Error bars denote standard deviation about the mean. Data points were derived from the average of at least six replicate cultures.

Conditioned media from the five prioritized LAB isolates were further tested against the six ESKAPE pathogens (*E. faecium*, *S. aureus*, *K. pneumoniae*, *A. baumannii*, *P. aeruginosa*, and *E.* spp.), as well as three common skin commensals (*Corynebacterium jeikeium*, *Staphylococcus hominis,* and *Staphylococcus epidermidis*) for their ability to inhibit planktonic growth. These tests provided a greater understanding of the range of activity of these LAB antimicrobials that can enable us to choose those that are best suited for potential medical applications. It was observed that when cultured with the six ESKAPE pathogens for 24 h, growth curve assays, and conditioned media from all five LAB were capable of inhibiting the growth of every ESKAPE pathogen tested (Figure 2A). In contrast, when conditioned media were added to the planktonic cultures of the skin commensals *C. jeikeium* (ATCC 43734), *S. hominis* (SK119), and *S. epidermidis* (SK135), growth inhibition was more variable (Figure 2B). *L. hilgardii* ATCC 27305 (also known as HMP 45) had the least impact on *S. epidermidis* planktonic growth among all three commensals tested. Due to this and the potential applications of antimicrobials derived from these supernatants, this *L. hilgardii* strain was chosen as the only LAB antimicrobial activity further characterized here.

### 2.3. L. hilgardii Antimicrobial Activity, Size Fractionation, and Thermostability

**Figure 3:**
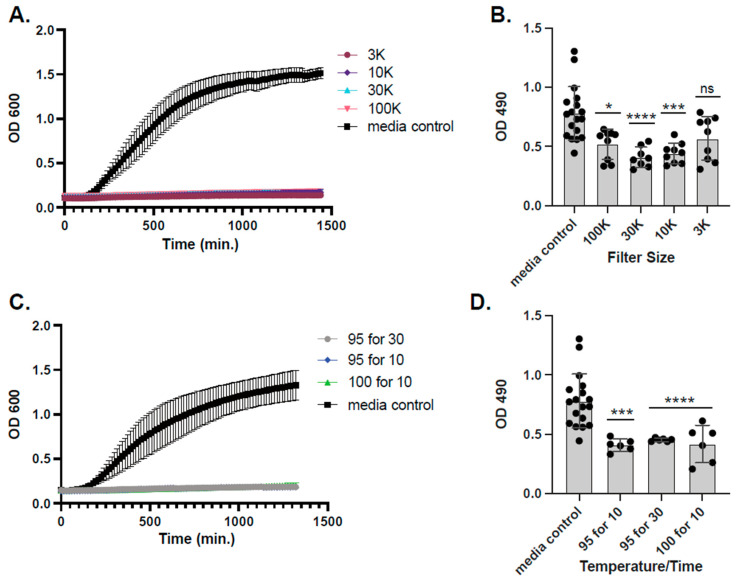
Inhibition by size-fractionated *Lentilactobacillus hilgardii* supernatants. *L. hilgardii* ATCC 27305 conditioned media was fractionated using Amicon Ultra centrifugal filters with a molecular weight cut-off of 100 kDa, 30 kDa, 10 kDa, or 3 kDa. Filtered conditioned media was co-incubated with *A. baumannii* in liquid BHI media for 24h to test for inhibition of planktonic growth (**A**) or dispersal pre-form biofilms (**B**). *L. hilgardii* ATCC 27305-conditioned media was also boiled, at 95 °C for 10 and 30 min, and 100 °C for 10 min, and tested for inhibition of planktonic growth (**C**) and dispersal of pre-formed biofilms (**D**). MRS is the media control. Experiments were performed in triplicate with error bars representing standard deviations. Black dots are values of individual replicates. For the non-parametric Kruskal–Wallis tests, asterisks denote the level of significance observed: ns, not significant; * *p* ≤ 0.05, *** *p* ≤ 0.001, and **** *p* ≤ 0.0001.

To understand whether a protein or a peptide was responsible for attenuation of *A. baumannii* planktonic growth, fractionation was carried out with Millipore Sigma Ultra-centrifugal filters. When *L. hilgardii*-conditioned media was fractionated as low as 3 kDa, no loss in planktonic inhibition of *A. baumannii* was observed (Figure 3A). When mature biofilms were treated with the filtered and conditioned media, only the media that passed through the 3 kDa filter lacked the ability to significantly reduce biofilm biomass (Figure 3B). Regardless of heat inactivation parameters tested (e.g., 95 °C for 10 or 30 min, or 100 °C for 10 min), significant planktonic killing and dispersal of *A. baumannii* pre-formed biofilms was never lost (Figure 3C,D).

### 2.4. Bioinformatic Prediction of L. hilgardii Antimicrobial Peptides

Since the observed antimicrobial activity fractionated at a size < 10 kDa, it seemed likely that at least some of the antimicrobial activity was due to an AMP [23]. To identify potential AMPs bioinformatically, *L. hilgardii* protein sequences were searched against a custom non-redundant comprehensive antimicrobial peptide database, CAMPdb, using BLASTP. This search produced 5 significant matches below our cut-offs (see Section 4), with 3 being peptides <100 aa in length and containing a diglycine leader sequence (Table 1). The other two CAMPdb matches were annotated as type B 50S ribosomal protein L31 and a putative holin-like toxin. One predicted peptide was an exact match to a predicted bacteriocin, AMPDB_45803, in the AMPDB [24] derived from the metagenomic sequence GenBank locus_id DUD35_04985 of an unknown *Lactobacillus* spp. within a medium-chain fatty acids-producing bioreactor [25]. Two additional *L. hilgardii* peptides (HMPREF0496_RS02405 and HMPREF0496_RS02410) had significant BLASTP matches to the class IIb *L. plantarum* NC8 peptides NC8α and NC8β, respectively [26].

**Figure 4:**
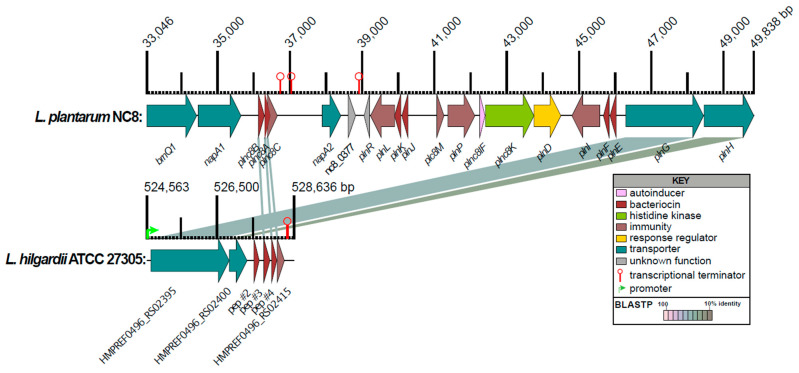
Comparison of predicted class II bacteriocins and surrounding genes from *L. hilgardii* ATCC 27305 to the *L. plantarum* NC8 *pln* operon. The linear map of protein-coding sequences (CDSs) is based on nucleotide sequences of the *L. plantarum* NC8 *pln* region (GenBank: AGRI01000003.1) and predicted CDSs from a region including predicted AMPs #2–4 in the HMP-sequenced genome of *L. hilgardii* ATCC 27305 (GenBank: NZ_GG669604). CDSs are labeled by gene symbol (*L. plantarum*) and by locus identifier or peptide designation (*L. hilgardii*) and colored by functional role categories of *L. plantarum* CDSs as noted in the boxed key. BLASTP matches between CDSs are colored by protein percent identity (see key). Green arrows and red hairpin structures indicate predicted promoters and transcriptional terminators, respectively. Numbers above major tick marks denote nucleotide coordinates in their respective GenBank accessions. Promoters were only displayed for *L. hilgardii*.

The genes encoding the predicted *L. hilgardii* AMPs were located next to each other in a cluster of 6 CDSs (HMPREF0496_RS02395 to HMPREF0496_RSRS02415). Class II AMPs also need to encode an ABC-type transporter (e.g., PlnG) and immunity proteins (e.g., PlnI) and an optional transporter accessory protein (e.g., PlnH) [27]. To validate the BLASTP results and to assign functions to the CDSs surrounding the *L. hilgardii* predicted AMPs, the *pln* operon from *L. plantarum* NC8 was compared to the 6-gene region in *L. hilgardii* (Figure 4). Just upstream of peptide #3 (HMPREF0496_RS02405) is a CDS encoding for a 48 amino acid peptide with a diglycine sequence, but no CAMPdb matches, which we refer to as peptide #2 (HMPREF0496_RS15215). An attempt was made to identify other potential diglycine leader-containing peptides by searching the protein sequences using the regex (i.e., regular expression in PERL programming vernacular) of “[LV][STDN]\S{2}[NAE]\S{3}[VITL]\SGG$ or [LVIM]\S{4}[LME]\S{2}[VITLPC]\SGG$”, leader sequences between 13 and 30 amino acids, and a full protein sequence length <100 amino acids. This resulted in 6 candidate peptides, including predicted peptides 1–4 (Table 1).

Since profile hidden Markov models (HMMs) are a sensitive and proven method to identify short sequence motifs based on multi-sequence alignments than simple pattern matching [28], we developed a novel HMM to identify the class II bacteriocin diglycine leader sequence and used the results of HMM searches to validate our BLASTP predictions and to identify other potentially novel candidate class II bacteriocins from the *L. hilgardii* proteome. The model was based on the alignment of 128 class II bacteriocin leader sequences (File S1) from the literature and AMP database sources (see Materials and Methods). A search of the *L. hilgardii* proteome using this new HMM (File S2), which we call JCVIFAM_00001, resulted in 4 matches above the inclusion threshold (Table 1). Only BLASTP-predicted peptides 1, 3, and 4 were also identified by our class II bacteriocin leader sequence HMM. The HMM also predicted HMPREF0496-RS15205, which was not identified by BLASTP. A search of the remaining LAB genomes of strains listed in Figure 1A resulted in the identification of 14 (9 not including *L. plantarum*) additional predicted class II bacteriocins (Table S2). No predicted class II bacteriocins were identified from six of the genomes.

### 2.5. Synthesis and Testing of Predicted L. hilgardii Peptides on A. baumannii Planktonic Cultures and Epithelial Cells

**Figure 5:**
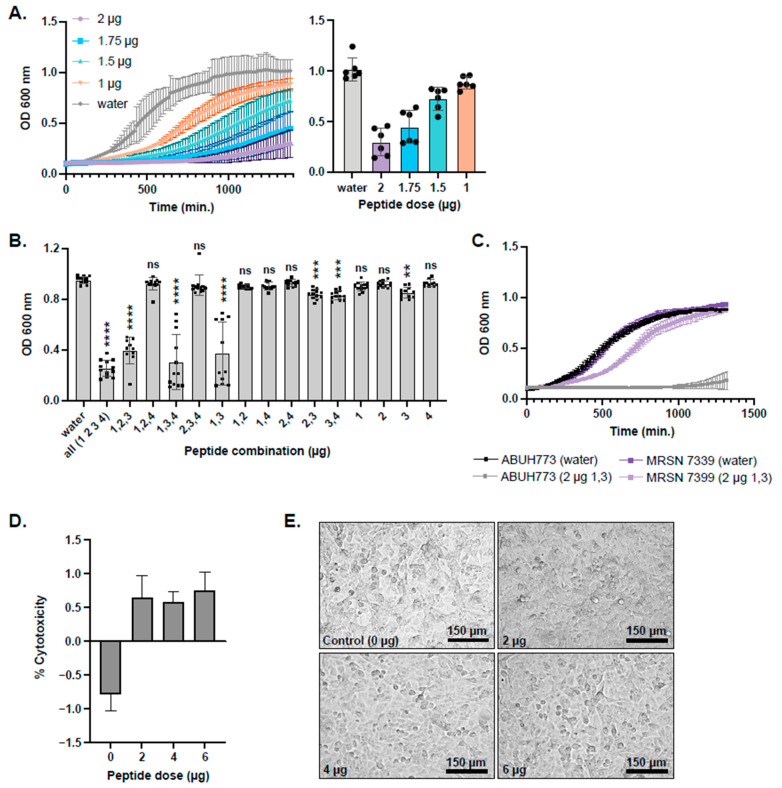
Testing of synthesized predicted peptides for activity and cytotoxicity. Chemically synthesized (85% purity) predicted antimicrobial peptides derived from bioinformatic analysis of the *L. hilgardii* genome were tested for MICs of planktonic 24 h growth of *A. baumannii* in broth cultures. All four peptides were pooled and added to 200 μL BHI media in the amounts shown in the key (**A**). Colors in (**A**) denote different peptide amounts or water (see key within left panel). It was found that 2 μg is the lowest amount of peptide required to strongly inhibit planktonic growth. The MIC of 2 μg (10 μg/mL) of each peptide was tested for inhibition of *A. baumannii* planktonic growth in the combinations noted on the X-axis (**B**). CRAb isolate MRSN 7339 was challenged with peptides 1 and 3 in 24 h growth curve assays (**C**). A549 cells were challenged with *L. hilgardii* antimicrobial peptides at doses of 2, 4, and 6 μg, and LDH release was assessed 4 h post-challenge (**D**). Light microscopy at 20× magnification of A549 cells challenged with the peptides at doses of 2, 4, and 6 μg shows no major cytopathic effect when compared to the control (**E**). Statistical differences relative to no peptide controls were determined by the Kruskal–Wallis test with Dunn’s multiple-comparison post-test. ** *p* ≤ 0.01, *** *p* ≤ 0.001, **** *p* ≤ 0.0001. Error bars denote standard deviation. Black dots are values of individual replicates.

Four predicted AMPs (peptides 1–4, Table 1) were synthesized at 85% purity by LifeTein LLC (Somerset, NJ, USA). Peptide 2 was included because it had a diglycine motif and was located just 5′ to the genes encoding peptides 3 and 4, despite not having a significant match (i.e., above the inclusion threshold) to JCVIFAM_00001 or BLASTP matches to our CAMPdb and lacking YGNG or GxxxG motifs. Peptide 5 was not included in this study because it lacked BLASTP matches to CAMPdb, was not adjacent to peptides 2–4 and lacked YGNG and GxxxG motifs despite the significant HMM profile match. It was observed that when co-incubated with equal proportions of the four synthesized peptides, *A. baumannii* planktonic growth was inhibited at a minimum inhibitory concentration (MIC) of 10 µg/mL (Figure 5A). Once the MIC was determined for the four-peptide cocktail, omission experiments were carried out to determine which peptide(s) were responsible for growth inhibition. These experiments showed that for a significant (*p* < 0.0001) attenuation in planktonic growth, only peptides 1 and 3 combined inhibited growth as well as all four peptides together (Figure 5B). Since ABUH773 is a GC2 isolate, we wanted to test peptides 1 and 3 for the ability to inhibit the growth of a different global clone. We tested peptides 1 and 3 for their ability to inhibit the planktonic growth of a GC1 isolate, MRSN 7339, which had a less pronounced effect on growth than the GC2 strain ABUH773 (Figure 5C). The respiratory tract is a frequent site for *A. baumannii* infection [29]. We tested the toxicity of peptides 1 and 3 together on human airway epithelial cells, which showed minimal toxicity of the peptides at concentrations that ablate *A. baumannii* growth (Figure 5D,E).

### 2.6. Testing L. hilgardii Peptides on ESKAPE Pathogens

Since conditioned media from *L. hilgardii* ATCC 27305 inhibited planktonic growth of the ESKAPE pathogen strains tested (Figure 2), we wanted to see if that activity was caused by peptides #1 and #3 at the same concentration that was effective on *A. baumannii* planktonic growth (10 µg/mL, Figure 5). Compared to conditioned media, which had an almost total inhibitory effect on the ESKAPE pathogens, the mix of peptides #1 and #3 showed an approximate 50% reduction in the growth of *E. faecium*, *S. aureus*, *K. pneumoniae*, *A. baumannii*, and *E. hormaechei* (Figure S1). Notably, the peptides did not exhibit activity against *P. aeruginosa*, whereas only the conditioned media inhibited growth (Figure S1).

### 2.7. Testing of Predicted L. hilgardii Peptides on A. baumannii Pre-Formed Biofilms

**Figure 6:**
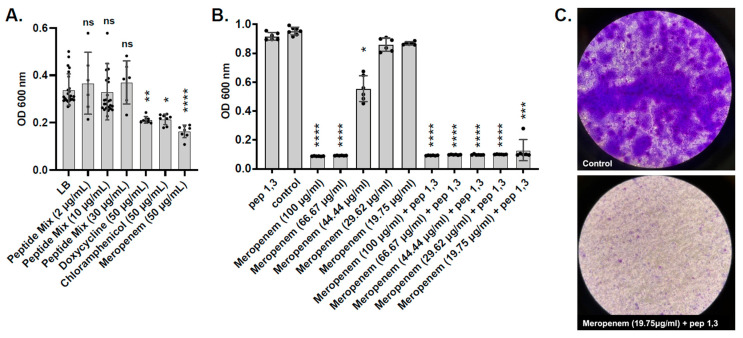
AMPs synergize with antibiotics against biofilms. *A. baumannii* biofilm biomass after treatment with 2, 10, or 30 μg/mL of the peptide mix or 50 μg/mL of Doxycycline, Chloramphenicol, or Meropenem (**A**). Biomass changes in biofilms treated with a combination of peptides 1 and 3 and Meropenem (**B**). Representative light microscopy pictures of crystal violet-stained mature *A. baumannii* biofilms. Control untreated and Meropenem (19.75 μg/mL) and peptide 1, 3 treated (**C**). Statistical differences relative to no peptide controls were determined by the Kruskal–Wallis test with Dunn’s multiple-comparison post-test. * *p* ≤ 0.05, ** *p* ≤ 0.01, *** *p* ≤ 0.001, **** *p* ≤ 0.0001. Error bars denote standard deviation. Black dots are values of individual replicates.

As with the ability to inhibit the planktonic growth of *A. baumannii*, *L. hilgardii*-conditioned media were also able to disrupt pre-formed biofilms, which is an important virulence determinant of *A. baumannii* and many other nosocomial pathogens. We therefore wanted to determine if a cocktail of all four synthesized peptides and common antibiotics was sufficient to disrupt pre-formed *A. baumannii* biofilms. We observed that peptides alone had no effect against biofilms at 2, 10, 30, or 200 µg/mL; however, 50 µg/mL of doxycycline, chloramphenicol, or meropenem was partially effective in reducing biofilm biomass (Figure 6A and Figure S2). Since meropenem had the highest effect against mature *A. baumannii* biofilms (Figure 6A), we tested its synergy with the AMPs. Addition of sub-MIC levels of meropenem between 19.75 and 44.44 μg/mL in combination with 10 µg/mL of peptides 1 and 3 significantly reduced the growth of a mature *A. baumannii* GC1 isolate MRSN 7339 biofilm (Figure 6B). Of note, combinatorial therapy led to a stark reduction in crystal violet-stained bacterial biofilms, suggesting that dispersal and decolonization can be achieved.

## 3. Discussion

In the search to combat the challenging biofilm formation of antibiotic-resistant bacteria such as *A. baumannii*, recent research has highlighted the potential of factors derived from commensal species of bacteria to both prevent biofilm formation and disrupt existing biofilms [21]. Conditioned media from various LAB species, including *L. hilgardii*, *L. buchneri*, *L. ruminis*, *L. fermentum*, and *L. antri*, demonstrated significant efficacy in both preventing the formation of and disrupting pre-formed *A. baumannii* biofilms. Further testing against ESKAPE pathogens and skin commensals revealed that conditioned media from these LAB could inhibit the growth of all ESKAPE pathogens, with variable effects on skin commensals. *L. hilgardii*-conditioned media were particularly noted for their ability to inhibit *A. baumannii* planktonic growth without complete inhibition of skin commensal growth. A cutting-edge bioinformatic approach was able to predict several antimicrobial peptides partially responsible for this activity. Synthesized peptides showed significant growth inhibition of *A. baumannii* and reduced growth of other ESKAPE pathogens. However, while these peptides were effective against planktonic cultures, they had no impact on pre-formed biofilms. Importantly, when combined with meropenem, the peptides demonstrated synergistic effects in reducing biofilm biomass. Together, our results provide a comprehensive approach to discover and characterize new antimicrobial peptides from commensal bacteria.

Current computational tools for identifying bacterial AMPs combine rich curated databases, motif/physicochemical scanners, and increasingly powerful machine-learning models. Large, actively maintained repositories such as APD3 [30] and CAMPR4 [31] provide the foundational datasets and basic search/prediction utilities that many pipelines still rely on. Classical predictors and web servers, like AMPA [32] and AntiBP2 [33], use residue-level scoring, charge/hydrophobicity filters, and handcrafted sequence features to locate short antimicrobial domains in proteins and peptides; however, AntiBP2 is now offline. Other bioinformatic resources, such as DBAASP [34] and AI4AMP [35], add species-specific activity prediction and improved physicochemical encodings, expanding from binary AMP/non-AMP calls toward estimated potency and target spectra; however, they either lack the ability to search whole genomes for AMPs or return too many predicted AMPs. Recently, deep-learning approaches such as iAMP-Attenpred [36], deepAMPNet [37], and deepAMP [38] have improved predictive performance and enabled the design of novel candidates; however, they are dependent on high-quality labeled data and require experimental MIC validation. Taken together, the current approaches focus on the identification of candidate bacterial AMPs; however, the best approach should aim to use targeted in silico methods coupled with wet-lab testing to improve prediction accuracy, species coverage, and reduce false-positive rates [39].

Despite the availability of AMP prediction tools, we were unsuccessful in using them to identify candidate AMPs for several reasons. As with many boutique online bioinformatic prediction resources, they fail to be maintained and are no longer available; therefore, we could not test all of them. The two that we tested (CAMPR4 and AI4AMP) on the proteome of *L. hilgardii* returned too many predicted AMPs to reasonably test. To be able to fine-tune peptide search parameters and have unprocessed (i.e., prepeptide or preform) sequences complete with leader sequences, we decided to download as many AMP databases that were still publicly available at the time of this project, as well as published AMPs with activity on *A. baumannii*, many of which were lacking from the public AMP databases. These peptide sequences were merged into a single, BLAST-formatted non-redundant comprehensive antimicrobial peptide database, CAMPdb. We also downloaded 179 AMP HMMs from the CAMPR4 website, which returned 5 matches using HMMSCAN. However, 4 of them were greater than 100 aa, and the one that was less than 100 aa had an E-value of 0.025 and did not have a diglycine leader motif. Using our CAMPdb, we extracted and aligned 128 diglycine-containing class IIa, IIb, and IId leader peptide sequences to build our new HMM, which outperformed existing HMMs for identifying class II bacteriocin leader peptide sequences from the genomes of LAB. Bagel4 [40], a web server and a standalone set of PERL scripts that identify ribosomally- synthesized and post-translationally modified peptides and bacteriocins, also contains 51 Pfam HMMs of bacteriocins and associated proteins (https://github.com/annejong/BAGEL4/tree/main/db_hmm, accessed on 31 August 2025). A search of these HMMs against the *L. hilgardii* genome resulted in two HMMs (PF10439 and PF03047) matching only peptides 1, 3, and 5 above the inclusion threshold. Despite having diglycine leader sequences and folding into alpha helices, peptides 2 and 4 were not identified by any of the 51 Pfams used by Bagel4, but JCVIFAM_00001 was able to. Further work will be needed to determine if peptides 2, 4, and 5 exhibit any activity towards Gram-negative or Gram-positive bacteria.

The AMPs identified, chemically synthesized, and tested in this study have characteristics typically found in class II bacteriocins, such as a short length (<60 amino acids), heat stability, a net positive charge, and the presence of a double or di-glycine leader sequence [27]. Because none of the peptides tested in this study were active alone and because they lack the signature “YGNGV” motif, they are likely not class IIa bacteriocins. However, since antimicrobial activity was only achieved with a pair of diglycine leader-containing and positively charged small peptides, peptides 1 and 3 match the description of class IIb bacteriocins, like PlnE/F and PlnJ/K. Both PlnE/F and PlnJ/K are two-peptide bacteriocins that consist of equal molar ratios of α (E and J) and β (F and K) amphiphilic alpha-helical peptide subunits that interact via “GxxxG” motifs to form the active bacteriocin that spans the target cell membrane, resulting in increased permeability to small molecules and ultimately cell death [27,41,42]. *L. hilgardii* mature peptides 1 and 3 have sequence lengths of 25 and 35 aa in length, which correspond to a size of 3.2 and 4.0 kDa, respectively. They are also thermostable up to 100 °C and have net positive charges of 6 and 5, respectively, all properties found in class II bacteriocins. Additionally, they both have predicted GxxxG-like (i.e., GxxxG/A/S/T) motifs, which promote binding of transmembrane domains of integral membrane proteins [43] and class IIb bacteriocins [41]. Peptide 1 has the sequence “GfkkT” near the C-terminus of the mature peptide, while peptide 3 has the sequence “GfnkG” also near the C-terminus (Table 1). Future work is needed to characterize the interactions of these two peptides and the role of these sequence motifs in this interaction.

Unlike typical class IIb two-peptide bacteriocins, whose gene encoding the prepeptides are next to each other, the genes coding for the pre-peptides of 1 and 3 are located in different operons, ~70 Kbp apart [41]. In *L. hilgardii* ATCC 27305, the genes encoding prepeptides 2, 3, and 4 are next to each other in a single operon along with predicted immunity and transport proteins (Figure 4). Peptide 4 has BLASTP similarity to Plantaricin NC8α, and Peptide 3 has similarity to Plantaricin NC8β, while peptides 1 and 2 have no discernible matches to two-peptide bacteriocins (Table 1). It could be that α and β subunits from different operons can mix and match as a mechanism to target different bacteria. Understanding the mode of action and the relationship between the interchangeability of α and β subunits and host range will require further investigation.

Challenges in disrupting biofilms compared to planktonic cells have been a hallmark of the war on AMR pathogens. The reduced efficacy of AMPs against bacterial biofilms is primarily due to the protective properties of the extracellular polymeric substance (EPS) matrix and adaptive resistance mechanisms within biofilm communities. The EPS matrix is mainly composed of polysaccharides, proteins, extracellular DNA, and lipids, and acts as a physical and chemical barrier that can sequester or neutralize AMPs, reducing their ability to reach target cells [44,45]. Positively charged EPS matrix components, such as the polysaccharide intercellular adhesin (PIA) in *S. epidermidis* and *S. aureus*, can electrostatically repel cationic AMPs such as LL-37, human β-defensin, and sequester both cationic and anionic AMPs, thereby restricting their access to bacterial cells under the EPS [46,47]. Another way biofilms can reduce AMP activity is the upregulation of countermeasures such as proteases. These can either degrade or modify the surface charge of AMPs, reducing their ability to bind to bacteria and lyse them [48]. We hypothesize that similar mechanisms are employed by *A. baumannii* to prevent AMP activity and that disrupting the matrix by their combination with antibiotics may provide an efficient way to counter such mechanisms.

The multifactorial defense mechanisms used by bacteria to diminish the bactericidal potential of AMPs in biofilm contexts compared to planktonic states are of great importance to the field. Future studies should target the identification of new peptides that enhance the activity of decolonizing agents and antibiotic treatments. In addition, current studies targeting the use of bacteriophages (or bacteriophage-derived polysaccharide depolymerases) that can travel across or destroy the EPS matrix may further the synergy of peptides and antibiotics [49].

## 4. Materials and Methods

### 4.1. Bacterial Strains and Culturing Conditions

*Acinetobacter baumannii* ABUH773 was employed as the model organism for these experiments. *A. baumannii* ABUH773 is a CRAb global clone 2 (GC2) clade F clinical isolate from 2015 [50,51]. *A. baumannii* MRSN 7339 is a MDR CRAb global clone 1 (GC1) clinical isolate from 2004 [52]. *A. baumannii* was always grown at 37 °C, 5% CO_2_, and was cultivated in Brain Heart Infusion broth for 18 h. LAB were grown on and in MRS agar and broth. Thirteen human commensal LAB isolates were screened for their antimicrobial properties in the prevention of *A. baumannii* biofilm production (Table S1). All were isolated and sequenced by the human microbiome project (HMP) [53], and stock cultures were obtained from BEI resources. LAB were grown on MRS media aerobically at 37 °C, 5% CO_2_, except for *Limosilactobacillus antri* DSM 16041 and *Ligilactobacillus ruminus* ATCC 25644, which were grown at 37 °C in a Coy anaerobic chamber filled with a combination of N_2_ and standard mixed gases (CO_2_-H_2_-N_2_). Non-LAB commensal isolates were grown on BHI at 37 °C, 5% CO_2_. Supernatants of all commensals were prepared as follows: 10 mL of broth culture was seeded with 100 μL from starter cultures grown for 18 h at 37 °C. Liquid cultures were then centrifuged at 4000 RPM for 10 min. The supernatant was saved and filtered through a 0.22 μm cellulose membrane filter (Corning, New York, NY, USA). Before centrifugation, 200 μL was saved and serially diluted, then plated to determine CFU/mL. ESKAPE pathogen strains included *Enterococcus faecium* DO, *Staphylococcus aureus* SA113, *Klebsiella pneumoniae* VA367 [54], *Acinetobacter baumannii* ABUH773 [50,51], *Pseudomonas aeruginosa* PA01 [55], and *Enterobacter hormaechei* Eh1 [56] while human skin commensal bacteria tested included *Staphylococcus epidermidis* SK135, *Staphylococcus hominis* SK119, and *Corynebacterium jeikeium* ATCC 43734 (Table S1). ESKAPE and commensal bacteria were grown in BHI at 37 °C, except that *C. jeikeium* was supplemented with 1% (vol/vol) Tween-80 (Fisher).

### 4.2. Planktonic Growth Assays

24 h growth rate experiments were conducted in 96-well plates. Overnight broth cultures were diluted 1:100 with BHI or BHI supplemented with 1% Tween-80, providing bacterial cells in early log phase for the growth curve assays. The diluted bacterial cultures were mixed 50:50 and co-incubated with the cell-free LAB supernatants, along with MRS as a control, in a total volume of 200 μL. Negative treatment controls consisted of bacteria grown in media only; blank wells with and without media were used to define the background absorbance. The 96-well plates were incubated at 37 °C in a SpectraMax microplate reader (Molecular Devices, San Jose, CA, USA), with optical density measured at 600 nm every 15 min over 24 h.

### 4.3. Biofilm Assays

Biofilm disruption and prevention assays were largely conducted in the same manner, with some exceptions. In biofilm prevention assays, *A. baumannii* overnight cultures were diluted 1:100 and inoculated in well plates with cell-free supernatant (experimental) and MRS broth (negative control). The plates were then incubated at 37 °C and 5% CO_2_ statically overnight. The next day, the contents of the wells were removed, and the *A. baumannii* biofilms were fixed with 100% methanol (incubated at 4 °C for 10 min), then stained with filtered 0.1% crystal violet (incubated at 4 °C for 10 min), and finally washed with PBS. Ethanol was added to the wells, and absorbance at 490 nm was measured with a Tecan infinite M1000 microplate reader (Tecan US, Morrisville, NC, USA). Slightly different, biofilm disruption assays were conducted over the course of three days. *A. baumannii* overnight cultures were diluted 1:100 and inoculated in well plates with MRS broth to incubate at 37 °C, 5% CO_2_ statically overnight. The next day, the contents were gently removed from the wells, and fresh BHI and MRS were applied to each well. On the third day, the contents were removed, and the supernatants were applied to experimental wells, along with fresh BHI, and MRS was applied to control wells. Plates were incubated overnight, and the next day, the biofilm biomass was measured as described previously (fixing, staining, Tecan). Additional biofilm assays were performed using a combination of peptides, polymyxin B, 70% ethanol, and LB media at pH 5.

### 4.4. Planktonic and Biofilm Viability

Planktonic and biofilm viability tests were completed following biofilm disruption assays. On the final day of the assay, in place of fixing and staining, 200 μL of the contents of the wells were removed and serially diluted. 10 μL were plated on BHI to determine CFU/mL counts of the planktonic cells present in the experimental and control wells. For biofilm viability, a sterile tip was employed to scrape the biofilms, then inoculate 200 μL of sterile PBS. This was then serially diluted, and 10 μL were plated on BHI to determine CFU/mL counts. All BHI plates were incubated overnight at 37 °C, 5% CO_2_, and isolated colonies were counted the following day.

### 4.5. Fractionation Assays

Cell-free supernatants were fractionated into four different sizes: 100 kDa, 30 kDa, 10 kDa, and 3 kDa. Amicon Ultra-centrifugal filters (MilliporeSigma, Burlington, MA, USA) were employed for fractionated supernatants. Cell-free LAB supernatants, as well as MRS for controls, were loaded into Ultra-Centrifugal filters and spun at 4000 RPM in a swinging-bucket rotor, 20 min for 3 kDa and 10 min for 100 kDa, 30 kDa and 10 kDa. Fractionated supernatants were then utilized to conduct both 24 h growth assays and biofilm disruption assays as described above.

### 4.6. Bioinformatics

Protein-coding sequences (CDSs) from the genome of *Lentilactobacillus hilgardii* subsp. *gravesensis* ATCC 27305 HMP ID 496 (GenBank: NZ_GG669709, Biosample: SAMN00001468) were searched for matches to a custom comprehensive and non-redundant antimicrobial peptide database using NCBI BLAST 2.11.0+ [57]. The database was constructed using amino acid sequences from 5 antimicrobial peptide database resources: BACTIBASE [58,59], APD3 [30], DRAMP 3.0 [60], dbAMP 2.0 [61], and AMPDB v1 [24], and 179 peptide sequences manually entered from a review article on *Acinetobacter*-targeting AMPs [19]. A combined total of 86,662 amino acid sequences were made non-redundant into a database of 32,865 by clustering with cd-hit [62] version 4.8.1 using default settings, which cluster amino acids with a sequence identity threshold of 0.9. Peptide sequences < 100 aa were retained, resulting in a final database of 12,616 antimicrobial peptide reference sequences, which will be referred to as the comprehensive antimicrobial peptide database, CAMPdb. This database was used as the subject in BLASTP searches against 2892 query CDSs from *L. hilgardii* ATCC 27305 with an E-value cut-off of 0.01. The resulting top matches were filtered to remove the strings “lysozyme” and “Helix-turn-helix” from the database matches and amino acid sequences > 99 aa.

To identify class II bacteriocin diglycine leader sequences within the genome of *L. hilgardii* ATCC 27305, a collection of full-length prototypical class II diglycine leader-containing peptides was obtained from literature [27] and the above-mentioned AMP databases that labeled the class II bacteriocins and contain full-length peptide sequences [24,60]. The leader sequences were extracted from 128 non-redundant class IIa/IIb/IId peptides and aligned with M-COFFEE using the T-COFFEE Multiple Sequence Alignment Server (https://tcoffee.crg.eu/, accessed on 1 September 2024) [63]. The FASTA alignment (File S1) was converted to Stockholm format using msaconverter V0.0.3 (https://github.com/linzhi2013/msaconverter, accessed on 1 September 2024) (command line: msaconverter -i alignment.fasta -o alignment.sto -p fasta -q stockholm -t protein), which uses Biopython [64]. An HMM profile model was generated (JCVIFAM_00001.hmm, File S2) using the hmmbuild program (command line: hmmbuild --amino -o hmmbuild_summary_JCVIFAM_00001 JCVIFAM_00001.hmm alignment.sto) from HMMER 3.3.2 [65]. Protein sequences from the genome of *L. hilgardii* ATCC 27305 were searched using this novel HMM with the hmmsearch program (command line: hmmsearch -o JCVIFAM_00001.out --tblout JCVIFAM_00001_table.out --max JCVIFAM_00001.hmm SAMN00001468.pep) also from HMMER 3.3.2. Matches to this HMM that were above the software-determined inclusion threshold cut-off were considered to be positive matches. This HMM was validated by searching against the full-length class IIa,b,d peptide database used in the construction of the HMM, as well as by searching the protein sequences derived from the *L. plantarum pln* locus of strains C11 and NC8, GenBank accession numbers X94434 and AF522077, respectively. This HMM was able to match 139 out of 142 peptides from the seed alignment and the known AMPs *plnA*, *plnEF*, *plnJK*, and *plnN* from *L. plantarum* C11 and *plnc8IF*, *plnEF*, *plnJK*, and *plnc8AB* from *L. plantarum* NC8.

A linear genetic map illustration comparing a predicted AMP-containing region from the genome of *L. hilgardii* ATCC 27305 with the characterized *pln* operon of *L. plantarum* NC8 from GenBank accession AGRI01000003 was generated using LinearDisplay.pl (https://github.com/JCVenterInstitute/LinearDisplay, accessed on 1 October 2025) (command line: LinearDisplay.pl -A frag.file -F gene_att.file -P pairs.file -y -nt > linear.fig) [66]. The pairs.file was derived from blastp 2.11.0+ all-vs-all searches of the illustrated protein sequences (command line: blastp -query pep.file -db pep.file -out blastp.out -evalue 0.01 -outfmt 6 -num_threads 4).

### 4.7. Inhibition of Planktonic Growth by Chemically Synthesized Peptides

The four bioinformatically predicted peptide sequences were chemically synthesized by LifeTein LLC (Somerset, NJ, USA) to a minimum of 85% purity at a quantity of 4 mg each. All peptides were resuspended in water. Minimum Inhibitory Concentration (MIC) experiments were conducted first to determine the threshold of these peptides and their hypothesized antimicrobial abilities. During MIC assays, all four peptides were used in conjunction, mixed at the same volume, and co-incubated with *A. baumannii* in 24 h assays (exactly as described above). The volume of peptides for MIC assays shown in this paper started at 2 µg and went down to 1 µg, decreasing by 0.25 µg. Initial MIC experiments began at 200 µg and decreased tenfold to 2 µg. After MIC assays, synergy tests were made with single omission, double omission, and triple omission experiments (SOE, DOE, and TOE, respectively).

### 4.8. Synergy Between Peptides and Antibiotics

The 24 h planktonic growth curve assay was used as described above. An overnight broth culture of *A. baumannii* MRSN 7339 was diluted 1:100 in 100 μL BHI supplemented with meropenem dilutions in 96-well plates. Five concentrations of meropenem, ranging from 100 to 19.75 μg/mL, were tested. Doxycycline and chloramphenicol were tested at a concentration of 50 μg/mL. Water or water containing 2 μg of each of peptides #1 and #3 was added to bring the culture up to 200 μL total volume.

### 4.9. Cytotoxicity Assay

The adenocarcinoma human alveolar basal epithelial cell line A549 was used. The A549 cell line was purchased from ATCC. Cell cultures were maintained in sterile filtered Dulbecco’s modified Eagle medium (DMEM) containing 10% fetal bovine serum and 1% Penicillin-Streptomycin at 37 °C with 5% CO_2_. For the LDH cytotoxicity assay, cells were seeded into half of a 96-well flat-bottom tissue culture-treated plate at 4 × 10^4^ cells per well in DMEM. After overnight incubation (~18 h) at 37 °C with 5% CO_2_, the DMEM culturing media was aspirated off and replaced with RPMI 1640 containing 2% fetal bovine serum. At this time, synthesized peptides #1 and #3 were added to the experimental wells at 3 set concentrations. A peptide master mix was prepared at a concentration of 1 µg (peptide)/1 µL using sterile molecular-grade water. This master mix was added to the experimental wells to make peptide doses of 2, 4, and 6 µg, resulting in final peptide concentrations of 10 µg/mL, 20 µg/mL, and 30 µg/mL, respectively. Each well was supplemented with water as needed to reach a final volume of 200 µL. After the addition of the peptide mixture, the plate was incubated at 37 °C with 5% CO_2_ for 24 h. LDH activity was measured using an Invitrogen™ CyQUANT LDH Cytotoxicity Assay Kit (Thermo Fisher Scientific, Waltham, MA, USA). Assay was conducted following the manufacturer’s protocol with a few adjustments. Maximum LDH activity controls wells were treated with 20 µL of Lysis Buffer. Prior to step 7, the plate was centrifuged at 400× *g* for 5 min at room temperature. Experimental results were obtained using a Tecan infinite M1000 (Tecan US, Morrisville, NC, USA) measurement of the assay plate at 490 nm and 680 nm. Data was analyzed and standardized using Microsoft Excel and Prism. This experiment was repeated three times.

### 4.10. Statistical Analysis

Unless otherwise noted, all experiments are composed of a minimum of three biological replicates, with ≥3 technical replicates each. Statistical comparisons were calculated using GraphPad Prism 8 (La Jolla, CA, USA). Comparisons between two cohorts at a single time point are calculated by Mann–Whitney U test. Comparisons between groups of >2 cohorts or groups given multiple treatments were calculated by ANOVA with Tukey’s (one-way) or Sidak’s (two-way) post-test or by Kruskal–Wallis H test with Dunn’s multiple comparison post-test, as determined by the normality of data groups. Repeated measures are accounted for whenever applicable.

## 5. Conclusions

This study provides a comprehensive platform for the discovery and characterization of AMPs and supports the notion of how commensals can be used to reduce, prevent, and decolonize biofilms from pathogenic bacteria in the community and nosocomial settings.

## Figures and Tables

**Table 1:** Predicted class II peptides containing double-glycine leader sequences.

Locus_id	Peptide	Amino Acid Sequence of Prepeptides *	Size (aa), (KDa), pI of Prepeptide	Size (aa), (Kda), pI of Mature Peptide	Charge, Hydrophobicity of Mature Peptide	Best BLASTP Match	BLASTP (id, E-Val)	Full Seq HMM (E-Val, Score)	Domain HMM (E-Val, Score)
		|-----------leader--------GG|▼|---------mature peptide--------|							
HMPREF0496_RS14880	1	MFNQEKENMSQRYEELSADELSHISGG VTRYRHHEKKSWIDDFMK***GFKKT***FC	52, 6.3, 7.4	25, 3.2, 10.2	6, 8.7	AMPDB_45803	100%, 3 × 10^−36^	1.5 × 10^−4^, 19.6	3 × 10^−4^, 18.6
HMPREF0496_RS15215	2	MSRNNLTILSTHKLVSVIGG QTFPIPNKPFGDRYPITIQPIIRNAYSF	48, 5.4, 10.8	28, 3.3, 10.1	2, 10.1	No match	N.A.	3.8 × 10^−2^, 12	4.8 × 10^−2^, 11.7
HMPREF0496_RS02405	3	MNKLSKFSKVTDKDLSRINGG GVWWTVITTIGKVGYSAYKDRNDIKS**GFNKG**FKKP	56, 6.3, 10.6	35, 4.0, 10.4	5, 8.4	BAC103 (Plantaricin NC8β)	49%, 2 × 10^−10^	2.5 × 10^−4^, 18.9	4.5 × 10^−4^, 18.1
HMPREF0496_RS02410	4	MKNIKVVKDLDLKAVTGG DWASPFWNSW**GYTQG**KKATWNLKHPFVRF	47, 5.5, 10.5	29, 3.6, 10.5	4, 10.6	BAC089 (Plantaricin NC8α)	47%, 1 × 10^−13^	1.6 × 10^−3^, 16.4	2.1 × 10^−3^, 16.0
HMPREF0496_RS15205	5	MKDNFKNLNSYKKLDVNSLNLIEGG NSVASQVSDIFSRFKRAFSGSFVYKVSGRNQF	57, 6.5, 9.4	32, 3.6, 11.1	4, 7.8	No match	N.A.	6.3 × 10^−4^, 17.6	1.2 × 10^−3^, 16.8

* Underscore, double-glycine motif; filled triangle, conserved cleavage site; bold, GxxxG motifs; bold italics, putative GxxxT motif. HMM results using JCVIFAM_00001.

## Data Availability

Data is contained within the article or Supplementary Material.

## Supplementary Materials

The following supporting information can be downloaded at: https://www.mdpi.com/article/10.3390/antibiotics14101004/s1, Table S1: Bacterial strains used in this study; Table S2: Predicted class II peptides containing double-glycine leader sequences from other LAB genomes; Figure S1: The effect of peptides 1 and 3 on ESKAPE pathogen planktonic growth; Figure S2: The effect of peptides 1 and 3 on *A. baumannii* biofilms at a high dose. File S1: Multi-sequence alignment of class II bacteriocin diglycine leader sequences; File S2: Class II bacteriocin diglycine leader sequence profile HMM JCVIFAM_00001.

## Abbreviations

The following abbreviations are used in this manuscript:

aa	Amino acid
AMPs	Antimicrobial peptides
AMR	Antimicrobial resistance
ATCC	American Type Culture Collection
BEI	Biodefense and Emerging Infections research
BHI	Bovine heart infusion
BLASTP	Basic Local Alignment Search Tool Protein
CAMPdb	Comprehensive antimicrobial peptide database
CDC	Centers for Disease Control and Prevention
CDSs	Coding sequences
CRAb	Carbapenem-resistant *A. baumannii*
DMEM	Dubecco’s Modified Eagle Medium
DNA	Deoxyribonucleic acid
DOE	Double omission experiment
EPS	Extracellular polymeric substance
ESKAPE	*E. faecium*, *S. aureus*, *K. pneumoniae*, *A. baumannii*, *P. aeruginosa*, and *Enterobacter* spp.
GC1	Global clone 1
GC2	Global clone 2
HAI	Healthcare-associated infections
HMM	Hidden Markov model
HMP	Human microbiome project
HMP ID	Human microbiome project identifier
Kbp	Kilobase pair
Kda	Kilodalton
LAB	Lactic acid bacteria
LDH	Lactate Dehydrogenase
LLC	Limited liability corporation
MIC	Minimum inhibitory concentration
min	Minute
mL	Milliliter
MRS	DeMan Rogosa Sharpe
nm	Nanometer
PIA	Polysaccharide intercellular adhesin
SOE	Single omission experiment
spp.	Species
TOE	Triple omission experiment
μL	Microliter
