# Supplementary material for: In Silico Identification and Molecular Characterization of Lentilactobacillus hilgardii Antimicrobial Peptides with Activity Against Carbapenem-Resistant Acinetobacter baumannii
Source: Antibiotics (Basel). 2025 Oct 10;14(10):1004. doi: 10.3390/antibiotics14101004 (PMC12561633; doi:10.3390/antibiotics14101004)
Supplement: Supplementary file 1 [file antibiotics-14-01004-s001.zip › TableS1.pdf]

Table S1. Bacterial strains used in this study

Organism	Strain_ID / Repository_ID	HMP_ID	BCM_ID	Biosample_ID
Acinetobacter baumannii	ABUH773	N.A.	N.A.	SAMN05238697
Acinetobacter baumannii	MRSN 7339	N.A.	N.A.	SAMN02906929
Corynebacterium jeikeium	ATCC 43734	297	N.A.	SAMN00001506
Enterobacter hormaechei	Eh1	N.A.	N.A.	SAMN10654518
Enterococcus faecium	ATCC BAA-472; DO; TEX16	351	77	SAMN00002237
Klebsiella pneumoniae	VA367	N.A.	N.A.	N.A.
Lacticaseibacillus paracasei subsp. paracasei	ATCC 25302; DSM 5622; JCM 8130; NCDO 151; R094	530	48	SAMN00001470
Lactiplantibacillus plantarum subsp. plantarum	ATCC 14917; JCM 1149; CGMCC 1.2437	531	57	SAMN00001478
Lactobacillus delbrueckii subsp. lactis	ATCC 12315; DSM 20072; NCDO 1438	5505	118	SAMN06046522
Lactobacillus helveticus	ATCC 15009; DSM 20075; CGMCC 1.1877	518	91	SAMN00139430
Lactobacillus jensenii	JV-V16; BEI HM-105	526	30	SAMN00001502
Lactobacillus ultunensis	DSM 16047; CCUG 48460; CIP 109908; LMG 22117	548	84	SAMN00001484
Lentilactobacillus buchneri	ATCC 11577	497	46	SAMN00001469
Lentilactobacillus hilgardii subsp. gravesensis	ATCC 27305	496	45	SAMN00001468
Lentilactobacillus hilgardii	ATCC 8290; DSM 20176	519	44	SAMN00001467
Ligilactobacillus ruminis	ATCC 25644	542	54	SAMN00001480
Limosilactobacillus antri	DSM 16041; CCUG 48456; LMG 22111	494	80	SAMN00001477
Limosilactobacillus fermentum	ATCC 14931; NCIB 11840	511	55	SAMN00001473
Limosilactobacillus vaginalis	ATCC 49540; DSM 5837; NCTC 12197	549	50	SAMN00001485
Pseudomonas aeruginosa	PAO1	N.A.	N.A.	SAMN02603714
Staphylococcus aureus	SA113; ATCC 35556	N.A.	N.A.	SAMN35547312
Staphylococcus epidermidis	SK135; BEI HM-118	797	N.A.	SAMN00008358
Staphylococcus hominis	SK119; BEI HM-119	799	N.A.	SAMN00001902
