# Supplementary material for: In Silico Identification and Molecular Characterization of Lentilactobacillus hilgardii Antimicrobial Peptides with Activity Against Carbapenem-Resistant Acinetobacter baumannii
Source: Antibiotics (Basel). 2025 Oct 10;14(10):1004. doi: 10.3390/antibiotics14101004 (PMC12561633; doi:10.3390/antibiotics14101004)
Supplement: Supplementary file 1 [file antibiotics-14-01004-s001.zip › TableS2.pdf]

Table S2. Predicted class II peptides containing double-glycine leader sequences from other LAB genomes

Organism+A2:AA2:E16	Biosampe_id	Sign. (PBF)*	Locus_id	Description	Amino acid sequence of prepeptides†	Full seq HMM (E-val, score)	Domain HMM (E-val, score)
Lactobacillus buchneri ATCC 11577	SAMN00001469	****	HMPREF0497_0549-GG669710	bacteriocin-type signal sequence	MFNQEKENMSQHYQELSAEELSHISGG VTRYRYHEKKSWIDDFMK**GFKKT**FC	6.2e-05, 20.8	0.00017, 19.4
Ligilactobacillus ruminis ATCC 25644	SAMN00001480	****	HMPREF0542_RS06945-NZ_GL833110	leucocin A/sakacin P family class II bacteriocin	MENKKKLTKADLAKVTGG SRYYGN**GVTCG**KHKCTVNW**GQAWT**CGVNRLANFGHGNC	0.00012, 19.4	0.00016, 19.0
Ligilactobacillus ruminis ATCC 25644	SAMN00001480	****	HMPREF0542_RS11225-NZ_GL833110	hypothetical protein	MENTRTTARRLTENELRKIYGG KRSAYDSTHLRLTKSKKYCIFHLFSKGCR	0.009, 13.5	0.02, 12.4
Limosilactobacillus fermentum ATCC 14931	SAMN00001473	****	N.D.	N.D.	N.D.	N.D.	N.D.
Limosilactobacillus antri DSM 16041	SAMN00001477	****	N.D.	N.D.	N.D.	N.D.	N.D.
Lentilactobacillus hilgardii ATCC 8290	SAMN00001467	***	HMPREF0519_RS13220-NZ_GG669992	bacteriocin	MSQHYQELSAEELSHISGG VTRYRYHEKKSWIDDFMK**GFKKT**FC	8.6e-05, 20.2	0.0004, 18.1
Limosilactobacillus vaginalis ATCC 49540	SAMN00001485	***	N.D.	N.D.	N.D.	N.D.	N.D.
Lactobacillus delbrueckii subsp. lactis DSM 20072	SAMN06046522	***	N.D.	N.D.	N.D.	N.D.	N.D.
Lacticaseibacillus paracasei subsp. paracasei ATCC 25302	SAMN00001470	**	HMPREF0530_RS07290-NZ_GG670154	bacteriocin	MTNLEDKELSQITGG FAFVIPVAAIL**GFLAS**DAWSHADEIAS**GAKTG**WEIANKYKIY	0.00083, 17.2	0.0017, 16.2
Lacticaseibacillus paracasei subsp. paracasei ATCC 25302	SAMN00001470	**	HMPREF0530_RS05165-NZ_GG670155	Blp family class II bacteriocin	MKDVSVLNVTELAQVTGG GIGKCIA**GTVGGGVLG**ALTG**GGLGS**KTKNLYVIGGLGIL**GGVGG**AIY**GASES**CF	0.0011, 16.9	0.0011, 16.9
Lacticaseibacillus paracasei subsp. paracasei ATCC 25302	SAMN00001470	**	HMPREF0530_RS14895-NZ_GG670154	bacteriocin	MELLNEKELAHVIGG KRKCPKTPFDNTPGAWFAHLILGC	0.0076, 14.2	0.0097, 13.8
Lacticaseibacillus paracasei subsp. paracasei ATCC 25302	SAMN00001470	**	HMPREF0530_RS08625-NZ_GG670153	hypothetical protein	MLENNLVLKNFSCIDDSELAESQGG IGVVAT**GVIIG**SLTVFLS**GVFSG**FTDGMTQEKKPATV	0.0084, 14.0	0.016, 13.2
Lactobacillus jensenii JV-V16	SAMN00001502	*	N.D.	N.D.	N.D.	N.D.	N.D.
Lactobacillus ultunensis DSM 16047	SAMN00001484	*	HMPREF0548_RS11160-NZ_GG693254	hypothetical protein	MQKLDRFVSVSNSELTQVVGG DNADYNM**GYETG**RDMRRTVNHIVRWWNQHFFTRREMN	0.0048, 14.4	0.0074, 13.8
Lactiplantibacillus plantarum subsp. plantarum ATCC 14917	SAMN00001478	ns	HMPREF0531_RS08625-NZ_GL379763	bacteriocin	MKIQIKGMKQLSNKEMQKIVGG KSSAYSLQMGATAIKQVKKLFKKWGW	7.1e-05, 20.6	7.1e-05, 20.6
Lactiplantibacillus plantarum subsp. plantarum ATCC 14917	SAMN00001478	ns	HMPREF0531_RS08650-NZ_GL379763	plnF two-peptide bacteriocin plantaricin EF subunit PlnF	MKKFLVLRDRELNAISGG VFHAYSARGVRNNYKSAVGPADWVISAVRGFIHG	0.00029, 18.7	0.00043, 18.2
Lactiplantibacillus plantarum subsp. plantarum ATCC 14917	SAMN00001478	ns	HMPREF0531_RS08590-NZ_GL379763	plnK two-peptide bacteriocin plantaricin JK subunit PlnK	MKIKLTVLNEFEELTADAEKNISGG RRSRKNGIGYAIGYAFGAVERAVLGGSRDYNK	0.0016, 16.3	0.0025, 15.8
Lactiplantibacillus plantarum subsp. plantarum ATCC 14917	SAMN00001478	ns	HMPREF0531_RS08655-NZ_GL379763	plnE two-peptide bacteriocin plantaricin EF subunit PlnE	MLQFEKLQYSRLPQKKLAKISGG FNRGGYNFGKSVRHVVDAIGSVAGIRGILKSIR	0.016, 13.2‡	0.034, 12.2‡
Lactiplantibacillus plantarum subsp. plantarum ATCC 14917	SAMN00001478	ns	HMPREF0531_RS08595-NZ_GL379763	plnJ two-peptide bacteriocin plantaricin JK subunit PlnJ	MTVNKMIKDLDVVDAFAPISNNKLNGVVGG GAWKNFWSSLRKGFYDGEAGRAIRR	0.077, 11.1‡	0.17, 10.0‡
Lactobacillus helveticus DSM 20075	SAMN00139430	ns	N.D.	N.D.	N.D.	N.D.	N.D.

*non-parametric Kruskal-Wallis tests from biofilm prevention assays, asterisks denote the level of significance observed: ****P ≤ 0.0001, ***P ≤ 0.001, **P ≤ 0.01, *P ≤ 0.05 and ns, not significant.

†underscore, double-glycine motif; filled triangle, conserved cleavage site; bold, GxxxG/S/T motifs

‡below inclusion threshold

N.D., Not Detected
