# Supplementary material for: In Silico Identification and Molecular Characterization of Lentilactobacillus hilgardii Antimicrobial Peptides with Activity Against Carbapenem-Resistant Acinetobacter baumannii
Source: Antibiotics (Basel). 2025 Oct 10;14(10):1004. doi: 10.3390/antibiotics14101004 (PMC12561633; doi:10.3390/antibiotics14101004)
Supplement: Supplementary file 1 [file antibiotics-14-01004-s001.zip › FigureS1.pdf]

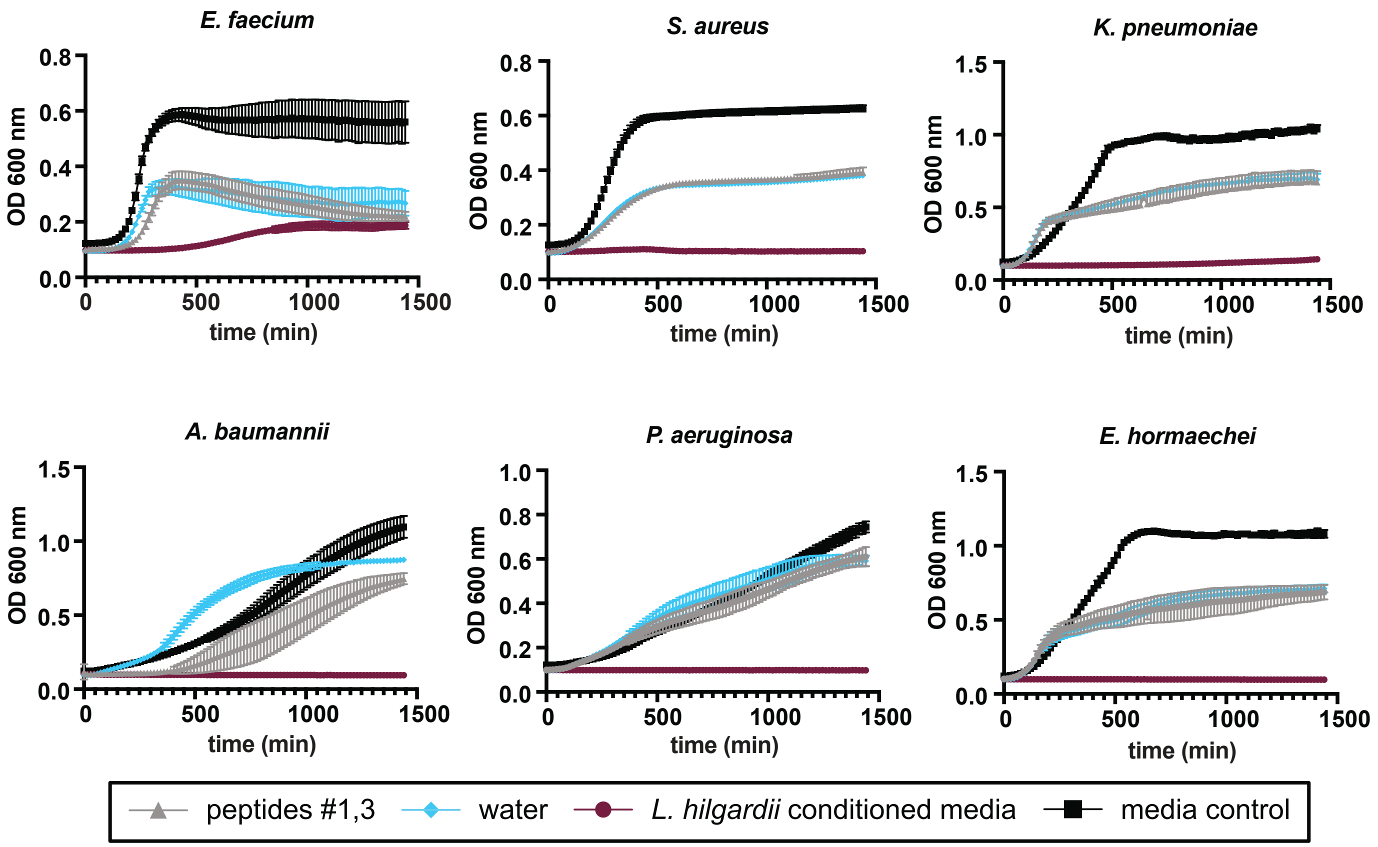


**Figure S1. The Effect of Peptides 1 and 3 on ESKAPE Pathogen Planktonic Growth.** ESKAPE pathogen cells were grown overnight in BHI broth then diluted 1:100 with BHI. Early log phase bacterial cultures were mixed with 2 µg each of peptides #1 and #3 (3.13 µM and 2.52 µM, respectively), along with the same volume of water as a negative control in a total volume of 200 µL. *L. higardii* conditioned media was also mixed 50:50 with cells with MRS as a media control in a total volume of 200 µL. The 96-well plates were incubated at 37°C in a microplate reader with optical density measured at 600 nm every 15 min over 24 h. Error bars denote standard deviation.
