# Supplementary material for: In Silico Identification and Molecular Characterization of Lentilactobacillus hilgardii Antimicrobial Peptides with Activity Against Carbapenem-Resistant Acinetobacter baumannii
Source: Antibiotics (Basel). 2025 Oct 10;14(10):1004. doi: 10.3390/antibiotics14101004 (PMC12561633; doi:10.3390/antibiotics14101004)
Supplement: Supplementary file 1 [file antibiotics-14-01004-s001.zip › FigureS2.pdf]

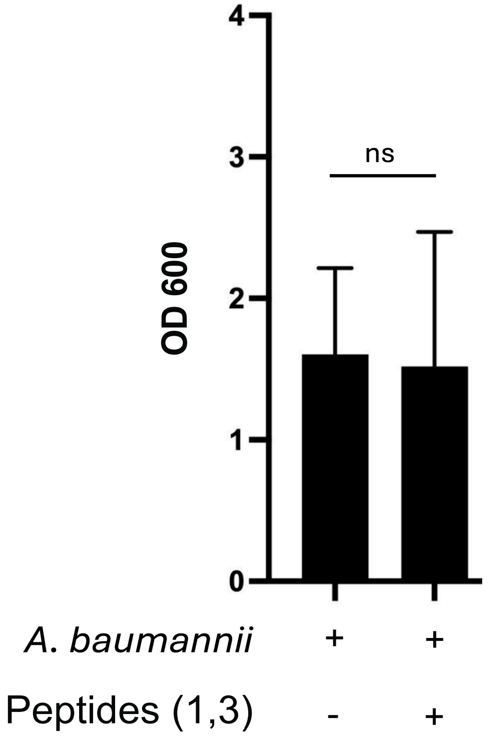

Figure S2. The effect of peptides 1 and 3 on *A. baumannii* biofilms at a high dose. *A. baumannii* biofilms were grown in culture media treated with 200 µg/mL of the peptide mix and stained with crystal violet for biomass assessment.
